# The relationship between early weight loss and weight loss maintenance with naltrexone-bupropion therapy

**DOI:** 10.1016/j.eclinm.2022.101436

**Published:** 2022-05-27

**Authors:** Carel W. le Roux, Nadège Fils-Aimé, Fernando Camacho, Errol Gould, Maxime Barakat

**Affiliations:** a Diabetes Complications Research Centre, Conway Institute, University College Dublin, Ireland; b Bausch Health, Laval, QC, Canada; c DAMOS Inc., Toronto, ON, Canada; d Currax Pharmaceuticals LLC, Brentwood, TN, United States of America

**Keywords:** Naltrexone/bupropion, Maintenance, Weight loss, COR-BMOD, COR-DM, COR-I, COR-II, LIGHT, IGNITE, AHEAD, Action in Health for Diabetes, BMI, body mass index, BMOD, behavior modification therapy, CBT, cognitive behavioral therapy, CI, confidence interval, CLI, comprehensive lifestyle intervention, COR, Contrave Obesity Research, DM, diabetes mellitus, ER, extended-release, MACE, major adverse cardiovascular events, NAFLD, non-alcoholic fatty liver disease, NB, naltrexone/bupropion, NWCR, National Weight Control Registry, SAEs, serious adverse events, SD, standard deviation, SMD, standardized mean difference

## Abstract

**Background:**

Extended-release (ER) naltrexone/bupropion (NB) was associated with greater weight loss than placebo in four randomized, 56-week trials. The association of NB with longer-term maintenance of weight loss remains unknown.

**Methods:**

We conducted a *post-hoc* analysis of four phase III, randomized, double-blind, placebo-controlled, 56-week studies (COR-I, COR-II, COR-BMOD, and COR-DM), the placebo-controlled cardiovascular outcomes trial LIGHT (208 weeks), and the randomized, open-label trial IGNITE (78 weeks). Included subjects were treated with NB 32 mg/360 mg or placebo, with baseline, week 16, and final time point data. The primary outcome was Kaplan-Meier-estimated weight loss maintenance in each study for up to 204 weeks.

**Findings:**

Our analysis included data from 10,198 particpants (NB=5412; placebo=4786). Proportions of patients with ≥5% or ≥10% weight loss maintenance were numerically higher for NB vs. placebo in all studies and time points. Differences were statistically significant for ≥5% weight loss maintenance in COR-BMOD and COR-I/-II at weeks 52 and 56 and the LIGHT study at weeks 52, 104, and 208. For ≥10% weight loss maintenance, differences were statistically significant in COR-I/COR-II at weeks 52 and 56.

**Interpretation:**

These data suggest that NB could be used as part of long-term, comprehensive weight loss and weight loss maintenance strategies.

**Funding:**

Orexigen Therapeutics, Inc. and Bausch Health Canada.

**Research in context**

**Evidence before this study**

We conducted a PubMed search for currently approved medications for weight loss and weight loss maintenance (liraglutide, naltrexone + bupropion, orlistat, and phentermine + topiramate). Clinical evidence suggests that all approved agents augment weight loss at one year; however, the quality and quantity of data showing longer-term weight loss maintenance is less robust.

**Added value of this study**

This is the first evaluation of longer-term (up to four years) weight loss maintenance with extended-release naltrexone/bupropion (NB) vs. placebo. The analysis was unique in that it compared weight loss maintenance with NB + lifestyle intervention among those who lost ≥5% of body weight with that regimen over 16 weeks vs. weight loss maintenance with placebo + lifestyle intervention among those who lost ≥5% of body weight with that regimen over 16 weeks. The ability of NB to promote weight loss maintenance was most evident in the COR-BMOD study, supporting the recommendation to include behavioral modification in comprehensive management strategies for obesity.

**Implications of all the available evidence**

These findings suggest that NB is an important intervention in the management of overweight and obesity, promoting clinically important weight loss that can be maintained for at least four years in most subjects.

Alt-text: Unlabelled box

## Introduction

Recent estimates by the World Health Organization suggest that approximately 13% of adults worldwide have a body mass index (BMI) of 30 kg/m^2^ or greater.^1^ The prevalence of obesity varies substantially across countries, from as low as 2·1% in Vietnam to as high as 61·0% in the South Pacific nation of Nauru.^2^ Prevalence estimates for obesity in the United States, Canada, and the United Kingdom are 36·2%, 29·4%, and 27·8%, respectively.^2^

Obesity is an established risk factor for type 2 diabetes, cardiovascular disease, cancer, and early mortality.^3^^,^^4^ Intentional weight loss of more than 10% in people with obesity is associated with decreases in all-cause, cardiovascular and cancer-related mortality^4^, ^5^, ^6^, ^7^, ^8^ and improvements in hyperglycemia, hypertension, and dyslipidemia.^4^, ^5^, ^6^ Furthermore, weight loss of more than 5% has been associated with reductions in the risk of developing type 2 diabetes and improvements in quality of life.^9^^,^^10^

Current guidelines for the management of obesity highlight a multifactorial approach that includes lifestyle modification (e.g., diet and physical activity), behavioral modification (e.g., cognitive behavioral therapy [CBT]), pharmacotherapy, bariatric surgery, or a combination of these.^11^ Clinical practice guidelines also stress that weight loss needs to be maintained for long-term health benefit.^11^, ^12^, ^13^, ^14^, ^15^ Indeed, fluctuation in body weight has been linked to increased mortality risk.^16^ The European Practical and Patient-centered Guidelines for Adult Obesity Management in Primary Care (2019) state that “preventing weight regain is the cornerstone of lifelong treatment, for any weight loss techniques used (behavioral, pharmaceutical treatments, or bariatric surgery).”^15^ According to the 2020 adult obesity guidelines in Canada, both lifestyle modification and pharmacotherapy play a role in weight loss maintenance.^11^

One of the currently approved pharmacotherapy regimens, extended-release (ER) naltrexone/bupropion (NB), has been associated with significant reductions in weight compared to placebo.^17^, ^18^, ^19^, ^20^, ^21^, ^22^ While the efficacy and safety of NB as an obesity medication has been established by these trials and led to its approval in the US, Canada, EU, and many other countries, its utility for weight loss maintenance has not been investigated.

To address this, we conducted a *post-hoc* analysis investigating the likelihood of maintaining weight loss with NB or placebo among patients who initially lost ≥5% or ≥10% of body weight and who were involved in the NB clinical development program.

## Methods

This *post-hoc* analysis of six NB trials included patients from four phase III, randomized, double-blind, placebo-controlled, 56-week studies (COR-I, COR-II, COR-BMOD, and COR-DM)^17^, ^18^, ^19^, ^20^; a multicenter, randomized, controlled, open-label trial (IGNITE)^21^; and a cardiovascular outcomes trial (LIGHT).^22^ Links to the six study protocols are available in the online publications for these studies, as well as via the link found in the respective ClinicalTrials.gov listing. For all studies included in the analysis, study participants provided written informed consent, and the protocols were approved by an institutional review board. The study complied with Good Clinical Practice standards.

The four 56-week studies formed the Contrave Obesity Research (COR) program, which evaluated the efficacy and safety of NB between March 2007 and June 2009. The primary findings were published in four separate publications between 2010 and 2013.^17^, ^18^, ^19^, ^20^ Three of these studies (COR-I, COR-II, and COR-BMOD) included subjects aged from 18 to 65 years, who had obesity (BMI 30–45 kg/m^2^) or overweight (BMI ≥27 kg/m^2^) and had dyslipidemia, controlled hypertension, or both.^17^, ^18^, ^19^ The fourth trial, COR-DM, included subjects aged 18 to 70 years with type 2 diabetes and a BMI of 27 to 45 kg/m^2^.^20^ Patients included in COR-DM also had glycated hemoglobin (HbA1c) of 7% to 10% and fasting blood glucose lower than 15 mmol/L (270 mg/dL).^20^

In COR-I, COR-II, and COR-DM, patients received periodic standard counseling recommendations on lifestyle modifications (hypocaloric diet [500 kcal per day deficit] and increased physical activity). Limited advice on behavioral modification was provided.^17^, ^18^, ^20^ In COR-BMOD, both the NB and placebo groups were counseled on an intensive program of diet, exercise, and behavior modification (BMOD) therapy.^19^ The BMOD components were 1) exercise: 180 min per week of vigorous physical activity for the first six months, increased to 360 min week for the subsequent six months; 2) individualized, balanced caloric deficit diet providing 15–20% of energy from protein, 30% or less energy from fat, and the remainder from carbohydrate; and 3) group (*n* = 10–20) counseling sessions every week for the first 16 weeks, bi-weekly for the next 12 weeks and monthly for the remaining seven months, up to 28 group sessions over the 56 weeks total, led by dietitians, behavioral psychologists or exercise specialists.^19^

IGNITE was a phase 3b, multicenter, randomized, open-label, controlled trial designed to assess the effects of NB in conjunction with a comprehensive lifestyle intervention (CLI) program compared with standard care (diet and exercise education and recommendations from the study site).^21^ The study included a total of 242 subjects aged 18 to 60 years who had obesity (BMI 30–45 kg/m^2^) or were overweight (BMI ≥27 kg/m^2^) and had dyslipidemia, controlled hypertension, or both. The controlled treatment period lasted for 26 weeks, after which subjects in the standard care group began receiving the NB + CLI intervention for the following 52 weeks. NB was initiated at 8/90 mg daily and escalated to 32/360 mg over the subsequent 3 weeks. CLI consisted of a telephone- and Internet-based progressive nutrition and exercise program with individualized goal setting and tracking tools. Subjects received up to 11 structured telephone calls from a coach or dietitian during the first 26 weeks and up to 12 additional calls over the uncontrolled treatment period. At baseline and week 10, subjects in the standard care group received instructions about exercise and hypocaloric diet (daily deficit 500 kcal). They were also given support tools, such as a nutrition tracker, a pedometer, and healthy weight literature. At week 16, if subjects in the NB/CLI group had not lost at least 5% of their initial body weight, or if they had an increase in systolic or diastolic blood pressure of 10 mmHg or more, they were discontinued from NB treatment.

LIGHT was a phase 3b, multicenter, randomized, double-blind, placebo-controlled trial to assess the occurrence of major adverse cardiovascular events (MACE) among men aged 45 years or older and women aged 50 years or older who were overweight or had obesity (BMI 27 to 50 kg/m^2^ and waist circumference of 88 cm or more for women and 102 cm or more for men) and had an increased risk of adverse cardiovascular outcomes.^22^ The prespecified definition of increased cardiovascular risk included documented pre-existing cardiovascular disease or type 2 diabetes plus two or more of hypertension, dyslipidemia requiring pharmacotherapy, high-density lipoprotein cholesterol lower than 1.30 mmol/L in women or lower than 1.04 mmol/L in men, or current tobacco smoking. Eligible subjects were randomized to either NB or placebo, titrated from 8/90 mg daily to 32/360 mg daily over the first four weeks of treatment, with an intended duration of randomized treatment between two and four years. All subjects were also encouraged (but not mandated) to participate in an Internet-based weight management program that included educational resources on healthy eating, exercise, and behavioral modifications. They also had access to a personal weight loss coach; programs to track weight, meals, and physical activity; and a low-fat, low-calorie meal plan. Subjects who did not lose 2% or more of their initial body weight or experienced a sustained increase in systolic or diastolic blood pressure of 10 mmHg or more during the first 16 weeks of randomized treatment were discontinued from the study medication. The prespecified primary outcome was time from treatment randomization to the first confirmed occurrence of a MACE (cardiovascular death, nonfatal stroke, or nonfatal myocardial infarction). Additional outcomes included changes in body weight, BMI, and waist circumference. This trial was terminated early after public release of confidential interim data. Although the planned assessment of cardiovascular safety was compromised, there remains a large body of data on long-term weight change and maintenance, which was used in the current analysis.

Body weight (assessed to the nearest 0.1 kg) was measured at each visit for all six studies. Height and waist circumference (in cm) were also measured in each study using the same methodology. Subjects treated with NB or placebo in the six studies and with baseline, week 16, and week 56 data (or data at baseline, week 16, and final time point if longer than 56 weeks) were pooled to constitute the overall population of the current analysis. Among NB-treated subjects, only those who were on the approved NB dose (ER naltrexone 32 mg/ER bupropion 360 mg) or placebo and who had a week 16 weight measurement were included.

The sub-populations of interest for the current analysis were subjects with a weight loss of ≥5% or ≥10% at week 16. The primary outcome for the analysis was maintenance of ≥5% or ≥10% weight loss in each of the responder populations at subsequent time points for each of the included studies. Weight loss maintenance at a time point *t* was defined as having maintained the threshold of weight loss (≥5% or ≥10% at week 16) at each time point measurement up to and including the time *t*. For example, subjects with ≥5% weight loss at weeks 16, 52, and 104, but not at week 56, were considered as having maintained ≥5% weight loss up to week 52. The Kaplan-Meier estimator was used to obtain point estimates and 95% confidence intervals (CIs) for the proportion of patients maintaining the weight loss up to each visit. Reported time points were week 52 (measured in all six studies), week 56 (measured in the four COR studies), week 78 (measured in IGNITE only), and weeks 104 and 208 (measured only in LIGHT). No weight imputation was done, and loss maintenance was based on data as observed. Because the inclusion criteria and designs for COR-I and COR-II were identical, the two studies were grouped together for this analysis. For each study, the log-rank test was used to determine the *p*-value for the comparison of the weight maintenance of the two treatments.

Safety was assessed by the incidence of serious adverse events (SAEs) among subjects treated with NB or placebo in each study. Safety was assessed using the overall population and the ≥5% and ≥10% responder populations. All subjects in this analysis had provided written informed consent as part of the inclusion process for the individual studies.

*Role of the funding source:* The funder had no role in the design and conduct of the study, collection, management, analysis and interpretation of the data. The authors and their contributions to the manuscript are independent from the funder. All authors had access to the data and contributed to the interpretation of study data, edit, review and approval of the final manuscript for submission.

## Results

**Figure 1:**
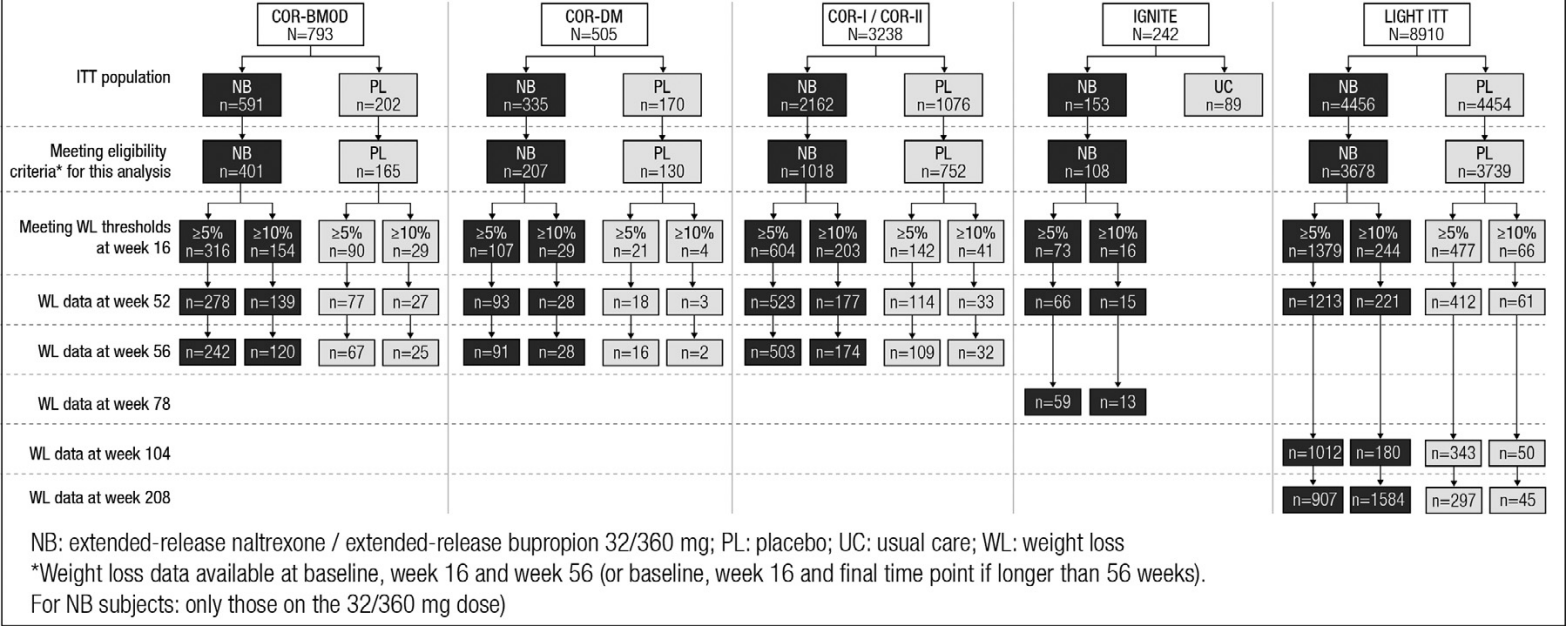
Patient Disposition. NB: extended-release naltrexone/extended-release bupropion 32/360 mg; PL: placebo; UC: usual care; WL: weight loss. *Weight loss data available at baseline, week 16, and week 56 (or baseline, week 16, and final time point if longer than 56 weeks). For NB subjects: only those on the 32/360 mg dose).

Data from six trials with a total of 10,198 patients (NB=5412; placebo=4786) was considered for the analysis. Baseline characteristics of the cohort by study and overall are shown in Table 1A. In total, 60·8% were female, and 83·1% were White/Caucasian. The average weight at baseline was 104·9 kg (SD, 18·6). The disposition of the patients who had lost ≥5% or ≥10% weight at week 16 across the subsequent time points is shown in Figure 1.

**Table 1:** Summary of particpants demographics.

A. All Subjects Eligible for This Analysis*								
		Study						SMD
		COR-BMOD	COR-DM	COR-I/COR-II	IGNITE	LIGHT	All	
Number of participants		566	337	1770	108	7417	10,198	
Sex, n (%)	Female	501 (88·5%)	174 (51·6%)	1469 (83·0%)	87 (80·6%)	3972 (53**·**6%)	6203 (60·8%)	0.481
	Male	65 (11·5%)	163 (48·4%)	301 (17·0%)	21 (19·4%)	3445 (46·4%)	3995 (39·2%)	
Mean age, years (SD)		46·1 (10·70)	54·3 (9·14)	44·7 (11·02)	47·6 (8·83)	61·1 (7·28)	57·1 (10·76)	0.898
Race, n (%)	White/Caucasian	424 (74·9%)	272 (80·7%)	1450 (81·9%)	88 (81·5%)	6240 (84·1%)	8474 (83·1%)	0.169
	Black/African American	107 (18·9%)	53 (15·7%)	254 (14·4%)	19 (17·6%)	1050 (14·2%)	1483 (14·5%)	
	Other or Unknown	35 (6·2%)	12 (3·6%)	66 (3·7%)	1 (0·9%)	127 (1·7%)	241 (2·4%)	
Mean weight at baseline, kg (SD)		101·0 (15·3)	106·0 (18·3)	100·1 (16·1)	102·2 (15·0)	106·3 (19·2)	104·9 (18·6)	0.205
Mean BMI at baseline, kg/m^2^ (SD)		36·5 (4·2)	36·5 (4·6)	36·1 (4·3)	36·4 (4·2)	37·3 (5·4)	37·0 (5·1)	0.108

B. Subjects with ≥5% Weight Loss at Week 16								
		Study						SMD
		COR-BMOD	COR-DM	COR-I/COR-II	IGNITE	LIGHT	All	
Number of participants		406	128	746	73	1856	3209	
Sex, n (%)	Female	360 (88·7%)	72 (56·3%)	630 (84·5%)	59 (80·8%)	975 (52·5%)	2096 (65·3%)	0.472
	Male	46 (11·3%)	56 (43·8%)	116 (15·5%)	14 (19·2%)	881 (47·5%)	1113 (34·7%)	
Mean age, years (SD)		46·7 (10·5)	56·1 (7·9)	45·2 (11·0)	47·7 (8·7)	61·6 (7·2)	55·3 (11·5)	0.957
Race, n (%)	White/Caucasian	323 (79·6%)	107 (83·6%)	652 (87·4%)	63 (86·3%)	1665 (89·7%)	2810 (87·6%)	0.233
	Black/African American	61 (15·0%)	19 (14·8%)	67 (9·0%)	10 (13·7%)	162 (8·7%)	319 (9·9%)	
	Other or Unknown	22 (5·4%)	2 (1·6%)	27 (3·6%)	0	29 (1·6%)	80 (2·5%)	
Mean weight at baseline, kg (SD)		100·2 (14·8)	104·8 (17·7)	99·0 (15·7)	101·8 (15·1)	105·8 (19·0)	103·4 (17·9)	0.217
Mean BMI at baseline, kg/m^2^ (SD)		36·2 (4·1)	36·6 (4·6)	35·9 (4·3)	36·2 (4·2)	37·3 (5·3)	36·8 (4·9)	0.144

C. Subjects with ≥10% Weight Loss at Week 16								
		Study						SMD
		COR-BMOD	COR-DM	COR-I/COR-II	IGNITE	LIGHT	All	
Number of participants		183	33	244	16	310	786	
Sex, n (%)	Female	161 (88·0%)	19 (57·6%)	209 (85·7%)	13 (81·3%)	154 (49·7%)	556 (70·7%)	0.489
	Male	22 (12·0%)	14 (42·4%)	35 (14·3%)	3 (18·8%)	156 (50·3%)	230 (29·3%)	
Mean age, years (SD)		46·6 (10·6)	56·1 (8·8)	45·3 (10·8)	50·6 (4·8)	61·4 (6·9)	52·5 (11·9)	1.001
Race, n (%)	White/Caucasian	159 (86·9%)	28 (84·8%)	225 (92·2%)	14 (87·5%)	284 (91·6%)	710 (90·3%)	0.291
	Black/African American	15 (8·2%)	5 (15·2%)	9 (3·7%)	2 (12·5%)	21 (6·8%)	52 (6·6%)	
	Other or Unknown	9 (4·9%)	0	10 (4·1%)	0	5 (1·6%)	24 (3·1%)	
Mean weight at baseline, kg (SD)		99·7 (16·0)	103·3 (18·6)	97·2 (15·7)	101·9 (16·5)	106·1 (18·8)	101·6 (17·6)	0.25
Mean BMI at baseline, kg/m^2^ (SD)		35·9 (4·1)	36·0 (4·4)	35·4 (4·1)	36·1 (4·6)	37·6 (5·4)	36·4 (4·8)	0.196

⁎ Weight loss data available at baseline, week 16, and week 56 (or baseline, week 16, and final time point if longer than 56 weeks). For NB subjects: only those on the 32/360 mg dose) COR: CONTRAVE Obesity Research; BMI: body mass index; BMOD: behavioral modification; DM: diabetes mellitus; SD: standard deviation; SMD: standardized mean difference.

**Figure 2:**
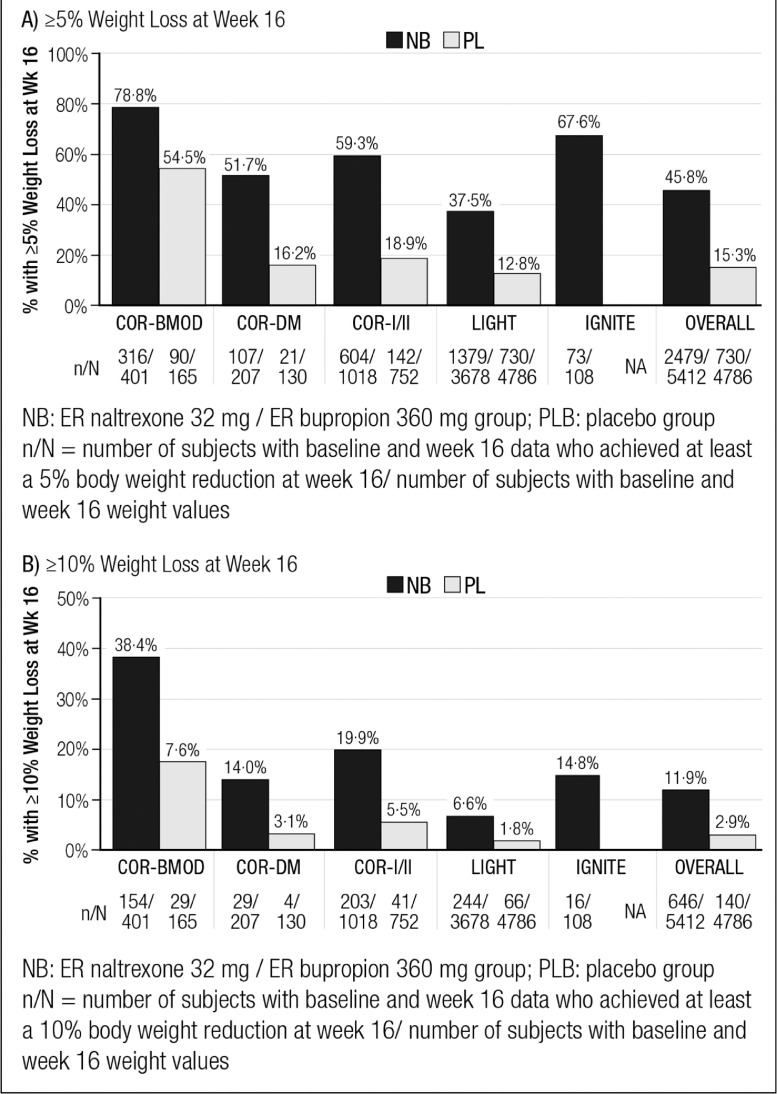
Proportions of Subjects Achieving Weight Loss Thresholds at Week 16: A) ≥5% Weight Loss; B) ≥10% Weight Loss (Observed).

At week 16, the proportions of patients with ≥5% weight loss were 45·8% (2479/5412) among those who received NB and 15·3% (730/4786) among participants in the placebo group (Figure 2A). The proportions of patients with ≥10% weight loss were 11·9% for NB (646/4786) and 2·9% for placebo (140/4786) (Figure 2B). Proportions of ≥5% and ≥10% responders varied across the studies (Figure 2). For the NB group, the proportion of patients with ≥5% weight loss ranged from 37·5% in the LIGHT study to 78·8% in the COR-BMOD trial. For the placebo group, the proportion of patients achieving ≥5% weight loss ranged from 12·8% in LIGHT to 54·5% in COR-BMOD. For the ≥10% threshold, values ranged from 6·6% (LIGHT) to 38·4% (COR-BMOD) for NB and from 1·8% (LIGHT) to 17·6% (COR-BMOD) for placebo. The baseline characteristics of patients with 5% and 10% weight loss are shown in Tables 1B and 1C, respectively.

**Figure 3:**
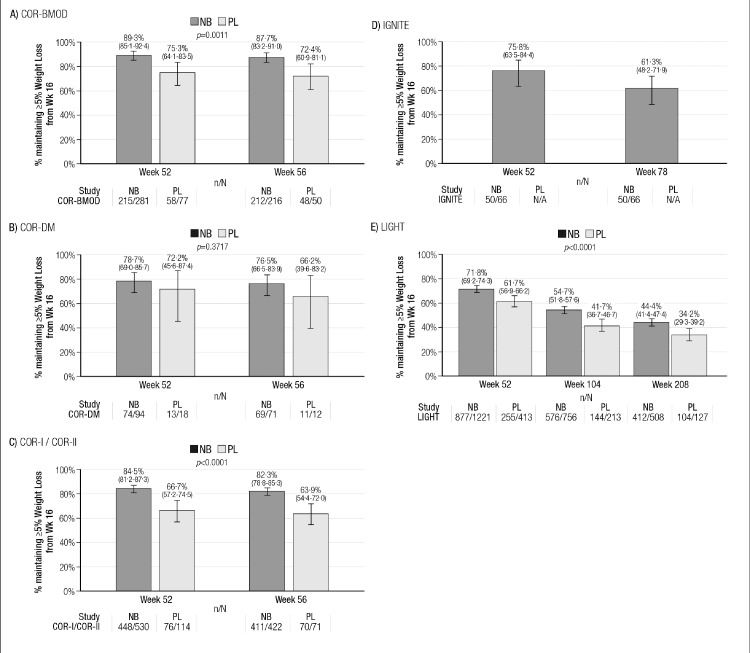
Proportions of Subjects With Maintenance of ≥5% Weight Loss, NB vs. PL, by Study (Kaplan-Meier Estimates with 95% CIs). Legend: Graphed values are Kaplan-Meier point estimates and 95% confidence intervals. NB: ER naltrexone 32 mg/ER bupropion 360 mg group; PLB: placebo group. n/*N* = number of subjects reporting ≥ 5% weight loss at the given visit/number of subjects reporting ≥ 5% weight loss at the previous visit and reporting weight loss at the visit. The Kaplan-Meier estimates the% of subjects with weight loss maintenance at the visit as the% of subjects with weight loss maintenance at the visit multiplied by the proportion of subjects with weight loss maintenance at the previous visit. For example, for COR-BMOD week 56, the proportion of subjects with 5% weight maintenance is equal to 100*(212/216)*0.893 = 87.7%. The p-values are the significance level of the Log-Rank test for testing treatment differences in the overall risk of losing weight maintenance.

**Figure 4:**
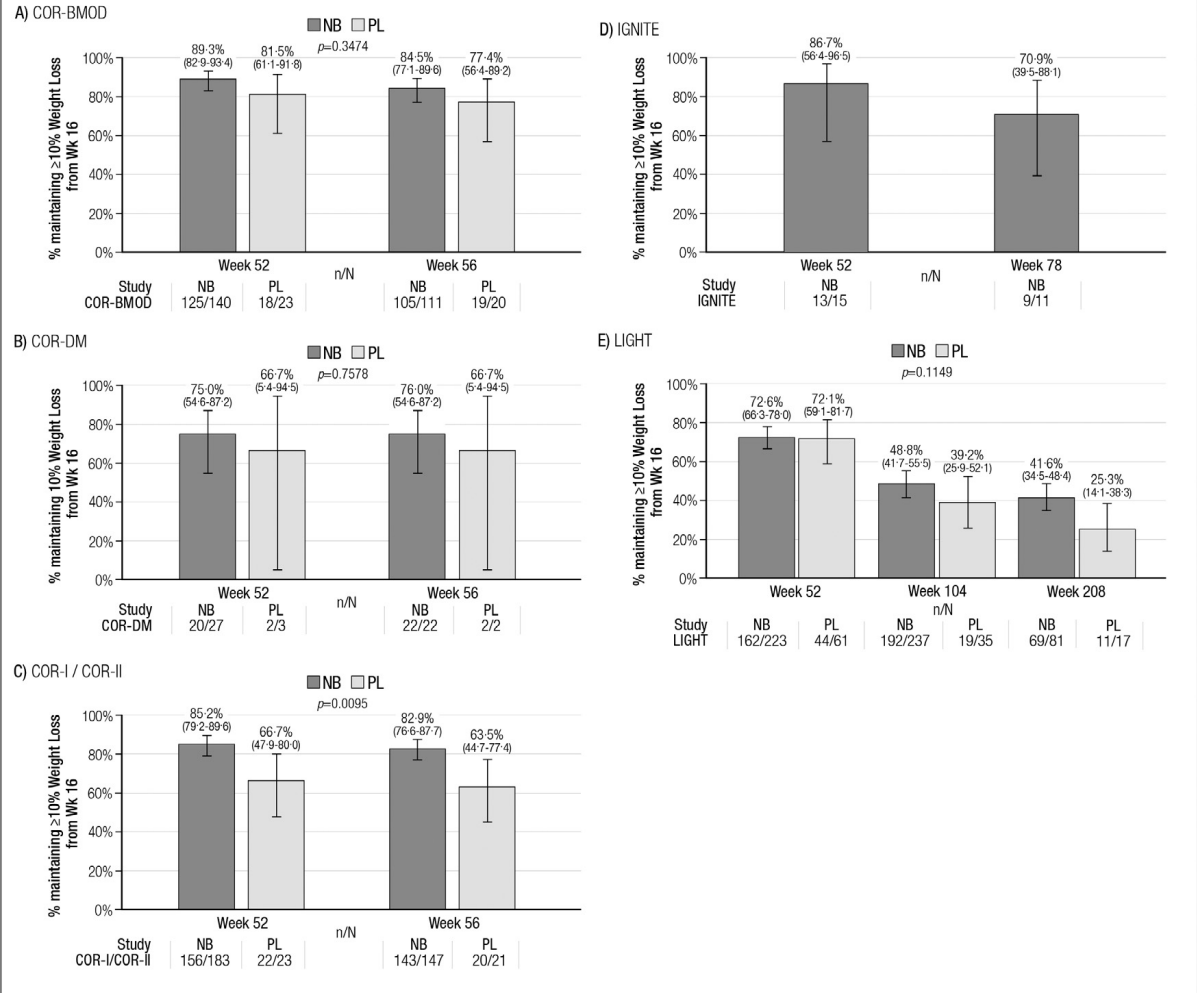
Proportions of Subjects With Maintenance of ≥10% Weight Loss, NB vs. PL, by Study (Kaplan-Meier Estimates, with 95% CIs). Legend: Graphed values are Kaplan-Meier point estimates and 95% confidence intervals. NB: ER naltrexone 32 mg/ER bupropion 360 mg group; PLB: placebo group. n/*N* = number of subjects reporting ≥ 10% weight loss at the given visit/number of subjects reporting ≥ 10% weight loss at the previous visit and reporting weight loss at the visit. The Kaplan-Meier estimates the% of subjects with weight loss maintenance at the visit as the% of subjects with weight loss maintenance at the visit multiplied by the proportion of subjects with weight loss maintenance at the previous visit. For example, for COR-BMOD week 56, the proportion of subjects with 10% weight maintenance is equal to 100*(105/111)*0.893 = 84.5%. The p-values are the significance level of the Log-Rank test for testing treatment differences in the overall risk of losing weight maintenance.

The Kaplan-Meier estimates of weight loss maintenance are shown in Figure 3 (≥5% weight loss maintenance) and Figure 4 (≥10% weight loss maintenance). With respect to ≥5% weight loss maintenance, the percentage of subjects with ≥5% weight loss maintenance was higher in the NB group than in the placebo group for all studies; this difference was statistically significant in the studies COR-BMOD, COR-I/COR-II and LIGHT (Figures 3A, 3C, and 3E). For the ≥10% weight loss maintenance Kaplan-Meier analyses, NB had numerically higher rates in each study; these were statistically significant in the COR-I/COR-II comparison (Figure 4C). In the LIGHT study, which had the longest follow-up, rates of ≥5% weight loss maintenance at week 208 were 44·4% for NB vs. 34·2% for placebo (*P*<0·001; Figure 3E). For ≥10% weight loss maintenance at week 208, the rates were 41·6% for NB and 25·3% for placebo (*P* = 0·11; Figure 4E).

SAEs were reported by 868 of the 5412 (16·0%) NB-treated patients and 831 of 4786 (17·4%) placebo-treated patients in the overall population (Table 2). The incidence of SAEs in the per-trial analysis varied from 0·9% (1/108) in the IGNITE trial to 22·2% (817/3678) in the LIGHT trial for NB and from 1·7% (13/752) in COR-DM to 21·7% (811/3739) in the LIGHT trial for placebo. The only SAE occurring in ≥1% of subjects in the overall population was osteoarthritis, with a 1·46% incidence (79/5412) among NB-treated subjects and 1·98% (95/4786) among placebo-treated subjects, and coronary artery disease among placebo subjects (1·19%; 57/4786).

**Table 2:** Serious adverse events with NB or placebo in the overall population, ≥5% responders and ≥10% responders.

Overall Population						
Treatment	COR-BMOD	COR DM	COR-I/COR-II	IGNITE	LIGHT	All
**NB**	19/401 (4.7%)	8/207 (3.9%)	23/1018 (2.3%)	1/108 (0.9%)	817/3678 (22.2%)	868/5412 (16.0%)
**Placebo**	0/165	7/130 (5.4%)	13/752 (1.7%)	NA	811/3739 (21.7%)	831/4786 (17.4%)
**≥5% Responder Population**						
**Treatment**	**COR-BMOD**	**COR DM**	**COR-I/COR-II**	**IGNITE**	**LIGHT**	**All**
**NB**	17/316 (5.4%)	6/107 (5.6%)	9/604 (1.5%)	1/73 (1.4%)	292/1379 (21.2%)	325/2479 (13.1%)
**Placebo**	0/90	0/21	2/142 (1.4%)	NA	112/477 (23.5%)	114/730 (15.6%)
**≥10% Responder Population**						
**Treatment**	**COR-BMOD**	**COR DM**	**COR-I/COR-II**	**IGNITE**	**LIGHT**	**All**
**NB**	8/154 (5.2%)	3/29 (10.3%)	6/203 (3.0%)	0/16 (0%)	50/244 (20.5%)	67/646 (10.4%)
**Placebo**	0/29	0/4	0/41	NA	9/66 (13.6%)	9/140 (6.4%)

Among individuals with ≥5% weight loss maintenance, the total incidence of SAEs across the studies was 13.1% (325/2479) in the NB group (ranging from 1.4% in IGNITE to 21.2% in LIGHT) and 15.6% in the placebo group (ranging from 1.4% in COR-I/COR-II to 23.5% in LIGHT). In the ≥10% responder population, the incidence of SAEs was 10.4% in the NB group (ranging from 0% in IGNITE to 20.5% in LIGHT) and 6.4% in the placebo group, with all SAEs occurring in the LIGHT trial.

## Discussion

In this first analysis of longer-term maintenance of weight loss with NB, we assessed the relationship between early weight loss and weight loss maintenance with naltrexone-bupropion therapy. Post-hoc analysis of six trials revealed that the proportions of individuals achieving ≥5% and ≥10% weight loss at week 16 were significantly higher in NB treatment groups than in placebo groups and that weight loss of ≥5% and ≥10% at week 16 of NB treatment was associated with a greater likelihood of weight loss maintenance up to 4 years. These findings support the clinical use of NB as a long-term weight loss and weight loss maintenance strategy.

Historical dogma suggested that it is relatively simple to lose weight but difficult to maintain weight loss.^23^, ^24^, ^25^ This sentiment was supported by an older meta-analysis of 29 studies (of lifestyle modifications alone) showing that within two years, more than half the weight initially lost was regained, and approximately 80% was regained within five years.^26^ However, recent evidence suggests that long-term weight loss maintenance is possible for a much larger proportion of individuals who lose substantial weight.^27^^,^^28^ Of the 887 Look AHEAD (Action in Health for Diabetes) study participants who lost ≥10% of their initial body weight over one year using lifestyle interventions, 374 (42%) maintained ≥10% weight loss at four years.^27^ Data from 2886 subjects in the National Weight Control Registry (NWCR) showed that individuals who had lost at least 13.6 kg (30 lbs) maintained this weight loss for at least one year.^28^ During prospective follow-up, most (87%) of these subjects were able to maintain at least 10% weight loss over a 10-year period. While methodologies differ substantially among weight loss and weight loss maintenance studies, the favorable results in recent studies may be attributable to more comprehensive weight loss and maintenance strategies and closer follow-up over time.

While experts agree that the goal is to maintain weight loss over the long term for optimal health benefits,^11^, ^12^, ^13^, ^14^, ^15^ there is no global consensus on the threshold of weight loss or the duration of maintenance that should be used to define sustained weight loss. In 2001, Wing and Hill suggested that successful weight loss maintainers should be defined as “individuals who have intentionally lost at least 10% of their body weight and kept it off at least 1 year”.^29^ Based on current international guidelines, this remains a reasonable consensus definition, although depending on the individual's initial weight and presence of obesity-related complications, some guidelines suggest that weight loss requirements may be lower. For example, in the European Practical and Patient-centered Guidelines for Adult Obesity Management in Primary Care (2019), the authors recommend targeting a sustained weight loss of 5–15% of initial body weight for individuals with type 2 diabetes, hypertension, dyslipidemia, or polycystic ovary syndrome to help prevent negative outcomes associated with these conditions.^15^ However, for individuals with non-alcoholic fatty liver disease (NAFLD), the goal is a weight loss of 10–40% to reduce intrahepatocellular lipids and inflammation.^15^

In our analysis, among individuals who had experienced substantial early weight loss with NB (at least 5% or at least 10% of body weight at week 16), NB added to lifestyle modifications was associated with greater proportions of subjects maintaining weight loss across subsequent time points for up to four years after the start of therapy. The proportion of NB-treated patients who maintained at least a 5% weight loss was 89% at week 52, 55% at week 104, and 44% at week 208. The differences in weight loss maintenance between NB and placebo groups were statistically significant for several of the individual trials included in the analysis.

Consistently, Fujioka et al.^30^ showed that among individuals who completed 1 year of NB treatment, ≥5% weight loss at week 16 was associated with an 11.7% weight loss at week 56 (1 year) and that most (85%) of these subjects had *a* ≥ 5% weight loss at week 56. Fujioka et al. conducted an integrated exploratory analysis of the four COR studies (COR-I, COR-II, COR-DM, COR-BMOD) to determine weight loss at 1 year for patients who lost at least 2%, 3%, 4%, and 5% of their initial body weight at weeks 8, 12, and 16. This analysis by Fujioka et al. aimed to determine the relationship between low weight loss thresholds (i.e., 2%, 3%, 4%, and 5%) and the likelihood of weight loss maintenance at 1 year, as well as the relationship between the time of initial weight loss (6, 12, or 16 weeks) and weight loss at 1 year. Although we also investigated the relationship between early weight loss and weight loss maintenance with NB therapy, in this study, we assessed the longer-term efficacy of NB. To this end, in addition to the four COR studies, we included data from the studies, LIGHT (NCT01601704) and IGNITE (NCT01764386), to determine the relationship between early weight loss and longer-term weight loss maintenance for up to four years (52, 56, 104, and 208 weeks). Fujioka et al. did not investigate the relationship between weight loss maintenance and initial weight loss with thresholds higher than the 5% threshold. In contrast, we explored the relationship between weight loss maintenance and initial (at week 16) weight loss of ≥5% and ≥10%. In addition to having a higher number of participants completing treatment for up to 52 weeks (5412 for NB, 4786 for placebo] in our study; 2073 [1310 for NB, 763 for placebo] in Fujioka et al.), we also assessed how long a weight loss of ≥5% and ≥10% could be maintained up to 4 years.

The NB clinical trial program demonstrated that the addition of NB to lifestyle modifications was associated with greater weight loss than lifestyle modifications alone.^17^, ^18^, ^19^, ^20^ The observations from this analysis suggest the greater ability of NB + lifestyle modification than lifestyle modification alone to help individuals maintain weight loss. Both of these observations are consistent with the recommendations of current clinical practice guidelines, which embrace a comprehensive approach to weight loss and weight loss maintenance that includes behavioral interventions, medication, surgery, or their combination to support lifestyle changes.^11^, ^12^, ^13^, ^14^, ^15^

Another aspect of this analysis that highlighted the importance of comprehensive management was that the proportion of subjects who achieved at least a 5% or 10% weight loss at week 16 were highest in the COR-BMOD trial (78·8% in the NB group and 54·5% in the placebo group) and lowest in the LIGHT study (37·5% in the NB group and 12·8% in the placebo group). Furthermore, both NB- and placebo-treated subjects in the COR-BMOD study had the highest weight loss maintenance rates (both ≥5% and ≥10%) among the included studies. It is worth noting that COR-BMOD incorporated a structured behavioral intervention in both treatment arms in line with the comprehensive guideline approach; LIGHT subjects had access to additional weight loss support (e.g., Internet-based tools and counseling), but these were not mandated. It is also worth noting that although NB treatment resulted in a significant early (at week 16) weight loss in COR-DM study subjects, NB had no significant effects on weight loss maintenance (week 52 or 56). Confounding factors, including the concomitant antidiabetic medications prescribed in the two groups and changes in those medications over time, could have affected weight loss in these patients. Medications for the treatment of dyslipidemia and hypertension were also allowed in the COR-DM study. Although doses of medications for dyslipidemia and hypertension were stable for at least 4 weeks prior to randomization, changes in those medications and their doses over time may have affected the ability of NB to promote weight loss in these patients.

Several aspects of this analysis differ from previously published analyses of weight loss maintenance with other weight loss medications. The SCALE Maintenance study randomized 422 individuals who had lost ≥5% body weight over four to 12 weeks with a low-calorie diet (1200–1400 kcal per day, including the daily use of up to three liquid meal replacements) to maintenance with liraglutide 3.0 mg or placebo once daily subcutaneously.^31^ Significantly more subjects maintained the ≥5% run-in weight loss with liraglutide compared with placebo over 56 weeks (81·4% vs. 48·9%; estimated odds ratio, 4·8; 95% CI, 3·0 to 7·7; *P*<0·0001). Other studies of weight loss medications have evaluated long-term weight loss, but not specifically weight loss maintenance among those who responded to a certain threshold at a given time point in the trial. In the SCALE Obesity and Prediabetes trial (*n* = 2254 with prediabetes and a BMI of at least 30 kg/m^2^, or at least 27 kg/m^2^ with complications), three years of treatment with liraglutide 3.0 mg or placebo subcutaneously once daily were associated with weight reductions of 6·1% and 1·9%, respectively (estimated treatment difference −4·3%, 95% CI, −4·9 to −3·7, *P*<0·0001).^32^

The weight loss effects of orlistat, a gastric and pancreatic lipase inhibitor, were also evaluated over the long term. The 4-year double-blind, randomized, placebo-controlled XENDOS trial randomized 3304 subjects with obesity to receive orlistat or placebo (each in addition to lifestyle changes).^33^ After 4 years, mean weight loss was 5·8 kg with orlistat and 3·0 kg with placebo (*P*<0·001).

The strengths of our analysis are the large number of patients included and the well-defined and diverse methodologies and populations of the component studies. The limitations include the inherent drawbacks of *post-hoc* studies, missing data from the subsequent time points of the analysis, and the relatively low number of individuals who had ≥10% weight loss at week 16, which limited the statistical power of our analysis to detect differences between NB and placebo in the comparisons of ≥10% weight loss maintenance. This refers to the power of detecting group differences in some of the studies because of the small sample size. For example, the COR-DM study has only 29 subjects in the treatment group and 4 in the placebo. With this sample size, the power of detecting a 8.3% difference between in these two groups is less than 50%. Another limitation of this study is that the population analyzed consisted predominantly of Caucasians, with a limited representation of Black/African American patients and patients of other or unknown races. Racial disparities in obesity and obesity treatment outcomes have been identified and reviewed extensively.^34^, ^35^, ^36^, ^37^ Therefore, this racial imbalance in the analyzed population renders the generalizability to other racial/ethnic groups unclear. Based on this small number of Black/African American patients, it appears that Black/African American patients had inferior weight loss outcomes, although this finding needs to be confirmed in a larger cohort. Similarly, the fact that women were largely overrepresented in some of the studies analyzed makes the generalizability to men unclear.

In this *post-hoc* analysis of weight loss maintenance using data from the NB clinical trial program, a substantial proportion of individuals who achieved ≥5% or ≥10% weight loss with NB at week 16 maintained a long-term weight loss up to 208 weeks. These proportions were consistently higher than the weight loss maintenance rates in placebo-treated individuals. These findings add to the growing evidence base suggesting that weight loss maintenance is feasible for most patients who achieve early weight loss targets and support current guideline recommendations to address obesity by striving for weight loss maintenance using a comprehensive approach.

### Data sharing statement

Data can be available upon reasonable request to the corresponding author.

### Funding

Orexigen Therapeutics, Inc. and Bausch Health Canada.

### Contributors

ClR and FC contributed to the conceptualization of the paper and methodology. MB was responsible for funding acquisition. FC was responsible for data curation and formal analysis. Project administration was primarily handled by NFA. ClR was responsible for writing the original draft. All authors contributed equally to the review and editing of the manuscript. ClR, MB, NFA, EG, and FC have accessed and verified the underlying data.

## Declaration of interests

ClR reports grants from Science Foundation Ireland, Health Research Board, Irish Research Council, during the conduct of the study; from NovoNordisk, GI Dynamics, personal fees from Eli Lilly, Sanofi Aventis, Astra Zeneca, Janssen, Bristol-Myers Squibb, Boehringer-Ingelheim, grants and personal fees from Johnson and Johnson, grants from AnaBio, other from Keyron, Neurovalence, outside the submitted work. ClR has been part of national and or global advisory boards for Consilient Health, Novo Nordisk, GI Dynamics, Herbalife, Keyron, Sanofi, and Boehringer Ingelheim. MB, and NFA are employees of, and shareholders in, Bausch Health Companies. FC received consulting fees from Bausch Health. EG is an employee of Currax Pharmaceuticals LLC during the conduct of this study, outside of the submitted work.

## References

[1] World Health Organization. Obesity and Overweight. Online at https://www.who.int/news-room/fact-sheets/detail/obesity-and-overweight. Accessed 3 April, 2020.

[2] World Health Organization. Global Health Observatory Data Repository. Online at https://apps.who.int/gho/data/node.main.A900A?lang=en. Accessed 3 April, 2020.

[3] Guh D.P., Zhang W., Bansback N., Amarsi Z., Birmingham C.L., Anis A.H. (2009). The incidence of co-morbidities related to obesity and overweight: a systematic review and meta-analysis. BMC Public Health.

[4] Magkos F., Fraterrigo G., Yoshino J. (2016). Effects of moderate and subsequent progressive weight loss on metabolic function and adipose tissue biology in humans with obesity. Cell Metab.

[5] Knowler W.C., Barrett-Connor E., Fowler S.E. (2002). Reduction in the incidence of type 2 diabetes with lifestyle intervention or metformin. N Engl J Med.

[6] Wing R.R., Look AHEAD Research Group (2010). Long-term effects of a lifestyle intervention on weight and cardiovascular risk factors in individuals with type 2 diabetes mellitus: four-year results of the look AHEAD trial. Arch Intern Med.

[7] Renehan A.G., Roberts D.L., Dive C. (2008). Obesity and cancer: pathophysiological and biological mechanisms. Arch Physiol Biochem.

[8] Williamson D.F., Pamuk E., Thun M., Flanders D., Byers T., Heath C. (1995). Prospective study of intentional weight loss and mortality in never-smoking overweight US white women aged 40–64 years. Am J Epidemiol.

[9] Fujioka K., O'Neil P.M., Davies M. (2016 Nov). Early Weight Loss with Liraglutide 3.0mg Predicts 1-Year Weight Loss and is Associated with Improvements in Clinical Markers. Obesity (Silver Spring).

[10] Chapter 17: Weight Management in Diabetes. Diabetes Canada Clinical Practice Guidelines. Eds. Wharton S, Pedersen SD, Lau D, Sharma A. Available at:https://www.diabetes.ca/health-care-providers/clinical-practice-guidelines/chapter-17#panel-tab_FullText Accessed 12 April, 2021.

[11] Wharton S., Lau D.C.W., Vallis M. (2020). Obesity in adults: a clinical practice guideline. CMAJ.

[12] National Institute for Health and Care Excellence (NICE). Obesity: identification, assessment and management. Clinical guideline [CG189]. Published date: 27 November 2014. Online athttps://www.nice.org.uk/guidance/cg189/chapter/About-this-guideline. Accessed 16 April, 2020.

[13] Moyer VA, U.S. Preventive Services Task Force (2012). Screening for and management of obesity in adults: U.S. preventive services task force recommendation statement. Ann Intern Med.

[14] Curry S.J., Krist A.H., US Preventive Services Task Force (2018). Behavioral weight loss interventions to prevent obesity-related morbidity and mortality in adults: US preventive services task force recommendation statement. JAMA.

[15] Durrer Schutz D., Busetto L., Dicker D. (2019). European practical and patient-centred guidelines for adult obesity management in primary care. Obes Facts.

[16] Oh T.J., Moon J.H., Choi S.H. (2019). Body-weight fluctuation and incident diabetes mellitus, cardiovascular disease, and mortality: a 16-year prospective cohort study. J Clin Endocrinol Metab.

[17] Greenway F.L., Fujioka K., Plodkowski R.A. (2010). Effect of naltrexone plus bupropion on weight loss in overweight and obese adults (COR-I): a multicentre, randomised, double-blind, placebo-controlled, phase 3 trial. Lancet.

[18] Apovian C.M., Aronne L., Rubino D. (2013). A randomized, phase 3 trial of naltrexone SR/bupropion SR on weight and obesity-related risk factors (COR-II). *Obesity* (Silver Spring).

[19] Wadden T.A., Foreyt J.P., Foster G.D. (2011). Weight loss with naltrexone SR/bupropion SR combination therapy as an adjunct to behavior modification: the COR-BMOD trial. *Obesity* (Silver Spring).

[20] Hollander P., Gupta A.K., Plodkowski R. (2013). Effects of naltrexone sustained-release/bupropion sustained-release combination therapy on body weight and glycemic parameters in overweight and obese patients with type 2 diabetes. Diabetes Care.

[21] Halseth A., Shan K., Walsh B., Gilder K., Fujioka K. (2017). Method-of-use study of naltrexone sustained release (SR)/bupropion SR on body weight in individuals with obesity. *Obesity* (Silver Spring).

[22] Nissen S.E., Wolski K.E., Prcela L. (2016). Effect of naltrexone-bupropion on major adverse cardiovascular events in overweight and obese patients with cardiovascular risk factors: a randomized clinical trial. JAMA.

[23] Mann T., Tomiyama A.J., Westling E. (2007). Medicare's search foreffective obesity treatments: diets are not the answer. Am Psychol.

[24] Mark A.L. (2006). Dietary therapy for obesity is a failure and pharmacotherapyis the future: a point of view. Clin Exp Pharmacol Physiol.

[25] Dulloo A.G., Montani J.P. (2015). Pathways from dieting to weight regain, to obesity and to the metabolic syndrome: an overview. Obes Rev.

[26] Anderson J.W., Konz E.C., Frederich R.C., Wood C.L. (2001). Long-term weight-loss maintenance: a meta-analysis of US studies. Am J Clin Nutr.

[27] Wadden T.A., Neiberg R.H., Wing R.R. (2011). Four-year weight losses in the look AHEAD study: factors associated with long-term success. Obesity (Silver Spring).

[28] Thomas J.G., Bond D.S., Phelan S., Hill J.O., Wing R.R. (2014). Weight-loss maintenance for 10 years in the national weight control registry. Am J Prev Med.

[29] Wing R.R., Hill J.O. (2001). Successful weight loss maintenance. Annu Rev Nutr.

[30] Fujioka K., Plodkowski R., O'Neil P.M., Gilder K., Walsh B., Greenway F.L (2016). The relationship between early weight loss and weight loss at 1 year with naltrexone ER/bupropion ER combination therapy. Int J Obes (Lond).

[31] Wadden T., Hollander P., Klein S. (2013). Weight maintenance and additional weight loss with liraglutide after low-calorie-diet-induced weight loss: the SCALE Maintenance randomized study. Int J Obes.

[32] le Roux C.W., Astrup A., Fujioka K. (2017). 3 years of liraglutide versus placebo for type 2 diabetes risk reduction and weight management in individuals with prediabetes: a randomised, double-blind trial. Lancet.

[33] Torgerson J.S., Hauptman J., Boldrin M.N. (2004). XENical in the prevention of diabetes in obese subjects (XENDOS) study: a randomized study of orlistat as an adjunct to lifestyle changes for the prevention of type 2 diabetes in obese patients. Diabetes Care.

[34] Kirby J.B., Liang L., Chen H.J., Wang Y. (2012). Race, place, and obesity: the complex relationships among community racial/ethnic composition, individual race/ethnicity, and obesity in the United States. Am J Public Health.

[35] Byrd A.S., Toth A.T., Stanford F.C. (2018). Racial disparities in obesity treatment. Curr Obes Rep.

[36] Cossrow N., Falkner B. (2004). Race/ethnic issues in obesity and obesity-related comorbidities. J Clin Endocrinol Metab.

[37] Wang L., Southerland J., Wang K. (2017). Ethnic differences in risk factors for obesity among adults in California, the United States. J Obes.

